# Sustained Low-Dose Treatment with the Histone Deacetylase Inhibitor LBH589 Induces Terminal Differentiation of Osteosarcoma Cells

**DOI:** 10.1155/2013/608964

**Published:** 2013-02-28

**Authors:** Jason E. Cain, Andrew McCaw, W. Samantha N. Jayasekara, Fernando J. Rossello, Kieren D. Marini, Aaron T. Irving, Maya Kansara, David M. Thomas, David M. Ashley, D. Neil Watkins

**Affiliations:** ^1^Centre for Cancer Research, Monash Institute of Medical Research, Monash University, 27-31 Wright St, Clayton, VIC 3168, Australia; ^2^Peter MacCallum Cancer Centre, St Andrews Place, East Melbourne, VIC 3002, Australia; ^3^Andrew Love Cancer Centre, Deakin University, Barwon Health, 70 Swanston St, Geelong, VIC 3220, Australia

## Abstract

Histone deacetylase inhibitors (HDACi) were identified nearly four decades ago based on their ability to induce cellular differentiation. However, the clinical development of these compounds as cancer therapies has focused on their capacity to induce apoptosis in hematologic and lymphoid malignancies, often in combination with conventional cytotoxic agents. In many cases, HDACi doses necessary to induce these effects result in significant toxicity. Since osteosarcoma cells express markers of terminal osteoblast differentiation in response to DNA methyltransferase inhibitors, we reasoned that the epigenetic reprogramming capacity of HDACi might be exploited for therapeutic benefit. Here, we show that continuous exposure of osteosarcoma cells to low concentrations of HDACi LBH589 (Panobinostat) over a three-week period induces terminal osteoblast differentiation and irreversible senescence without inducing cell death. Remarkably, transcriptional profiling revealed that HDACi therapy initiated gene signatures characteristic of chondrocyte and adipocyte lineages in addition to marked upregulation of mature osteoblast markers. In a mouse xenograft model, continuous low dose treatment with LBH589 induced a sustained cytostatic response accompanied by induction of mature osteoblast gene expression. These data suggest that the remarkable capacity of osteosarcoma cells to differentiate in response to HDACi therapy could be exploited for therapeutic benefit without inducing systemic toxicity.

## 1. Introduction

Osteosarcoma is a malignant mesenchymal neoplasm characterized by primitive osteoblastic cells [1] that represents the most prevalent primary tumour of bone, mainly arising in adolescents and in adults over the age of 50 [2]. Despite advances in surgical techniques and neoadjuvant chemotherapy, it remains the second leading cause of cancer-related death in children and young adults, and it contributes significantly to the health care burden of our society [3]. Approximately 20% of patients present with metastases and of the remaining 80%; a further 25%–50% will develop metastatic disease during their treatment [4, 5]. The use of adjuvant chemotherapy in osteosarcoma has significantly increased the 5-year survival rate from 10% to 70% for nonmetastatic disease [6]. However, cure rates for patients with metastatic or relapsed disease are poor, with a 5-year survival rate of <20% [7, 8]. The stagnation of these survival rates since the introduction of adjuvant chemotherapy three decades ago highlights the urgent need for new and improved therapeutic approaches to treat this disease.

Epigenetics is defined as a heritable change in gene expression without alteration of the underlying genetic sequence [9]. Epigenetic gene silencing is a critical modulator of key mammalian biological processes during development and has emerged as a central component of most cancers. Chromatin remodeling represents a major epigenetic mechanism of gene transcriptional regulation and is dependent on the posttranscriptional modification of histone proteins. Histone acetylation by histone acetyltransferases (HAT) results in the loosening of chromatin allowing replication and transcription, whereas deacetylation by histone deacetylases (HDAC) results in condensation of chromatin and transcriptional silencing. Deregulation of the intricate balance of these opposing functions is associated with different human diseases, including cancer. 

Histone deacetylase inhibitors (HDACis) are an emerging class of anticancer agents. HDACis preferentially alter the acetylation profile of both histone and nonhistone proteins in tumor cells leading to changes in gene expression, induction of apoptosis, and cell cycle arrest [10]. Whilst HDACi were originally discovered by their ability to induce erythroid differentiation of erythroleukemia cells [11, 12], the subsequent use of HDACi in cancer therapy has concentrated on its functions as a cytotoxic agent. The US Food and Drug Administration approval of the HDACi's vorinostat and romidepsin in 2006 and 2009, respectively, for the treatment of refractory cutaneous T-cell lymphoma has paved the way for the introduction of at least 10 other HDACis in human clinical trials [13]. While these studies demonstrate single-agent activity of HDACi in hematological malignancies, the effectiveness of HDACi in solid malignancies has been underwhelming [13]. Moreover, the significant toxicities associated with achieving a cytotoxic-related tumour response, particularly in solid tumours, is a major cause for concern [13].

Evidence that small molecules could induce epigenetic reprogramming was first described in mesenchymal stem cells, where the demethylating agent 5-Azacytidine induces terminal myoblast differentiation due to expression of the bHLH transcription factor MyoD [14]. Since similar effects have been observed in response to DNA demethylating agents in osteosarcoma cells *in vitro* [15], we determined whether HDACi had the capacity to act as a differentiation agent rather than a cytotoxic agent in osteosarcoma.

## 2. Materials and Methods

### 2.1. Cell Culture 

Authenticated B143, MG-63, Saos-2, SJSA, and U2OS human osteosarcoma cell lines were obtained from ATCC and maintained in DMEM (Gibco, Invitrogen) supplemented 10% FCS, 100 U/mL penicillin, and 10 mg/mL streptomycin in a humidified 5% CO_2_/95% air atmosphere at 37°C.

### 2.2. Compounds

LBH589 was provided by Novartis Pharmaceuticals (Basel, Switzerland). For *in vitro* and *in vivo* studies, LBH589 was reconstituted in DMSO and 5% dextrose, respectively, according to manufacturer's instructions.

### 2.3. Cell Viability

Cells (2 × 10^3^) were plated in 96 wells, allowed to adhere for 4 hrs, then treated for 96 hrs with increasing concentrations of LBH589 or DMSO vehicles. Relative viable cell number was determined using a bioreductive fluorometric assay (alamarBlue, Life Technologies, Invitrogen) according to manufacturer's directions. Fluorescence was determined after 6hrs using a microplate reader (FLUOROstar OPTIMA, BMG Labtech) set to 560 excitation/590 emission settings. Cell viability was measured at 0, 24, 48, and 96 hrs. Following each analysis, media containing residual alamarBlue reagent was removed and replaced with fresh treatment media allowing for repeated measurement of the same wells over the culture period. 

### 2.4. Analysis of Cell Cycle and Apoptosis by Flow Cytometry

Following 48 hrs culture in DMSO vehicle, 15 nM LBH589, or 200 nM LBH589, adherent and supernatant cells were harvested, washed twice with PBS and fixed in 70% ethanol at −20°C. Cells were then washed twice with PBS, and resuspended at a density of 1 × 10^6^ cells/mL in 20 *μ*g/mL propidium iodide/0/1% Triton X-100 staining solution with 2.5 *μ*g/mL RNase A. Cell cycle distribution was determined using the BD Biosciences FACSCanto II Analyzer. At least 20,000 cells were collected. The cell cycle profiles were determined using FlowJo software (version 7.6.3). For analysis of apoptosis, adherent and supernatant cells were harvested, washed twice in PBS, and resuspended in 1x binding buffer at a density of 1 × 10^6^ cells/mL. FITC Annexin V (BD Pharmingen) and propidium iodide at a final concentration of 50 *μ*g/mL was added to 1 × 10^5^ cells, gently vortexed, and incubated at room temperature in the dark for 15 mins. FITC Annexin V positive cells were analyzed using the BD Biosciences FACSCanto II Analyzer within 1 hr.

### 2.5. Qualitative and Quantitative Analysis of Differentiation

Cells (1 × 10^4^) were plated in 6-well plates, allowed to adhere overnight, and treated with 15 nM LBH589 or DMSO vehicle for 21 days. Media were changed every 3 days, and cells were split at 80% confluence. For qualitative analysis of osteoblast, adipocyte, and chondrocyte differentiations, cultures were washed in PBS, fixed in 10% buffered formalin, rinsed with dH_2_O, and stained in Alizarin Red (Sigma, 2%, pH4.1), Oil Red O (Sigma), and Alcian Blue (Sigma), respectively. For quantitative analysis of osteoblast differentiation, RNA was prepared using RNeasy Mini Kits (QIAGEN) and cDNA generated using first-strand cDNA synthesis (Invitrogen) from total RNA. Real-time PCR was carried out using SYBR Green (Applied Biosystems) according to manufacturers instructions using an ABI-Prism 7000 Sequence Detection System. All primers sequences are listed in Supplemental Table  1 in Supplementary Material available online at http://dx.doi.org/10.1155/2013/608964.

### 2.6. Senescence *β*-Galactosidase Staining

Following 21 days cultured cells were rinsed in PBS, fixed in lacZ fix solution (0.2% glutaraldehyde, 2% buffered formalin), washed in PBS, and incubated in staining solution (1 mg/mL X-gal, potassium ferrocyanide, and potassium ferricyanide) at 37°C overnight in the dark.

### 2.7. Protein Acetylation

Cells (1 × 10^6^) were plated in T25 flasks, allowed to adhere overnight and treated with 5, 10, 20, 50, 100, and 200 nM LBH589 or DMSO vehicle for 24 hrs. Cells were collected and centrifuged at 1000 rpm for 5 mins, washed in PBS, centrifuged as before, and resuspended in 100 uL of RIPA lysis buffer including protease inhibitors. Cells were incubated on ice for 30 mins, centrifuged at 15,000 rpm for 15 mins at 4°C, and the supernatant was collected in a clean tube. Protein acetylation was determined by western blot analysis on whole cell extracts, separated on a 4–12% Tris-Bis gel (Invitrogen), and transferred to nitrocellulose membrane (Perkin-Elmer). Blots were stained with anti-rabbit Acetyl-Histone H3 (Cell Signalling, no.9675)), anti-rabbit Acetyl-Histone H4 (Millipore, no. 06-598), anti-rabbit Acetyl-*α*-Tubulin (Cell Signalling, no. 5335), anti-rabbit Acetyl-p53 (Cell Signalling, no.2570S), and anti-mouse Actin (Abcam, ab3280) and visualized using the Odessey Infrared Imaging System (v3.0, LI-COR).

### 2.8. Clonogenic Assay

Following 21 days culture in 15 nM LBH589 or DMSO vehicle, 1000 cells were plated into 0.7% Noble agar (Becton, Dickinson and Company) in DMEM supplemented with 10% FCS, 100 U/mL penicillin, and 10 mg/mL streptomycin, atop of a 1% Noble Agar layer in a 35 mm culture plate. Normal growth media were added on top of the 0.7% agar layer once set and incubated for 28 days in a humidified 5% CO_2_/95% air atmosphere at 37°C. The top growth media were replaced every 3-4 days. At completion of the culture period, colonies were stained with 0.005% crystal violet, and colonies >500 *μ*m were counted under a dissecting microscope. Experiments were performed in triplicate.

### 2.9. Global Gene Expression Analysis

U2OS, SJSA, and B143 cells were collected from T25 flasks following 21 days culture in the presence of 15 nM LBH589 or DMSO vehicle. Cultures were performed in triplicate. RNA from cells was isolated using the RNeasy Mini Kit (QIAGEN) and snap frozen. Microarray analysis of RNA were performed by the Australia Genome Research Facility (Melbourne, Australia) using the HumanHT-12 v4 Expression BeadChip (Illumina), which contains 47,231 probes representing over 20,000 genes. Image processing and probe quantification was performed using the GenomeStudio software package (version 2011.1, Illumina, Inc., San Diego, CA, USA). Background correction was performed by subtracting the average value of the signals of built-in array negative controls, and background-corrected probe intensities were normalized using the cubic spline method. Normalized probe intensities were imported into Partek Genomics Suite (6th ed. Partek Inc. St. Louis, MO, USA) for differential gene expression and gene ontology analyses. An ANOVA test was performed and orthogonal contrasts were generated to compare different experimental groups (2-fold change; false discovery rate cutoff 0.05; step-up procedure [16]; *P* < 0.001). Gene ontology analysis was performed on the differentially expressed (1.2-fold change; false discovery rate cutoff 0.05; step-up procedure [16]; *P* < 0.001) of the U2OS cell line. Enriched genes for each of the assessed functional groups were determined by comparing the ratios of statistically significant differentially expressed genes in a functional group over all significant differentially expressed genes versus all genes in the functional group over all genes in the microarray chip (Fisher's exact test).

### 2.10. Human Osteosarcoma Xenograft Model

Female BALB/c nude mice (Animal Resources Centre, Australia), 6–8 weeks of age, received right flank injections of 1 × 10^6^ U2OS cells/mouse in 200 *μ*L of 1 : 1 mixed cell suspension and Matrigel. Tumour size was measured using digital calipers, and volumes were calculated according to the formula: Tumour volume (mm^3^) = (Width^2^ × Length)/2. Once tumours reached a volume of 100 mm^3^, animals were randomized to receive daily intraperitoneal injections of vehicle control (5% dextrose) or 2 mg/kg, 5 mg/kg, or 10 mg/kg LBH589. Mouse body weight and tumour volumes were measured daily. Experiments were terminated upon a ≥10% reduction in body weight; tumour size exceeded 10 mm in any axis, or after 17 days treatment. All experiments involving animals were approved in advance by an Animal Ethics Committee at Monash University and were carried out in accordance with “*Australian Code of Practice for the Care and Use of Animals for Scientific Purposes.*”

### 2.11. Statistics

Statistical analysis was performed using GraphPad Prism software (version 5.0c). Data were analyzed using 2-tailed Student's *t* test. A probability of less that 0.05 was considered to indicate statistical significance. 

## 3. Results

### 3.1. LBH589 Inhibits Human Osteosarcoma Cell Growth

**Figure 1:**
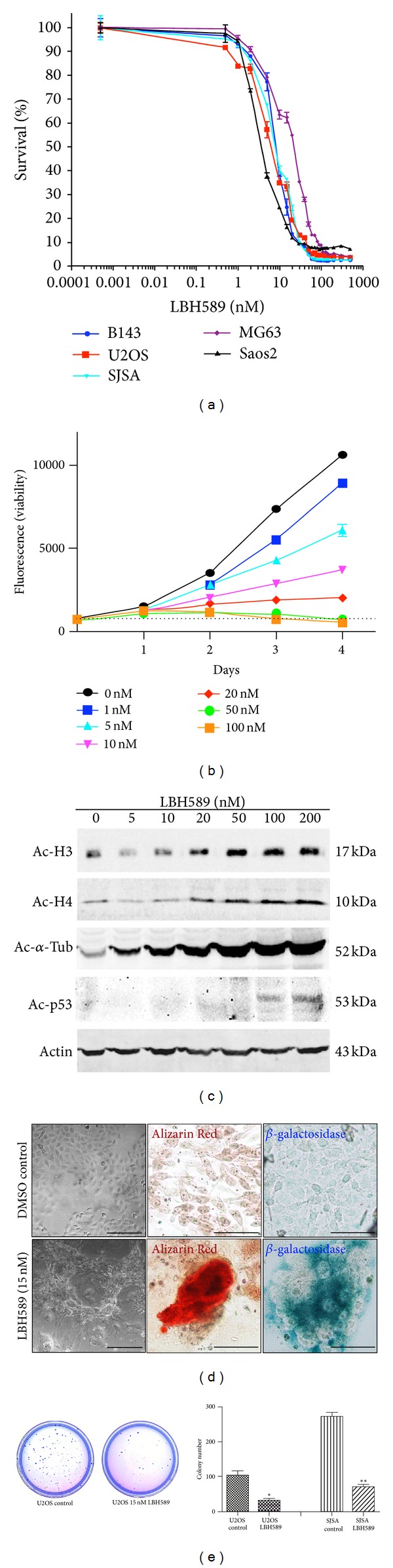
LBH589 inhibits cell growth and drives osteoblast differentiation of U2OS human osteosarcoma cells. (a) Survival curve of human osteosarcoma cell lines treated with increasing concentrations of LBH589. Data represent means ± SEM of quadruplicates. (b) Cell viability of U2OS cells treated with increasing concentrations of LBH589. Data represent means ± SEM of quadruplicates and dotted line = starting cell number. (c) Western blot analysis of U2OS cells with increasing concentrations of LBH589 for 24 hours. (d) Analysis of cell morphology, mineralized extracellular matrix deposition, and cellular senescence in U2OS cells treated for 21 days in 15 nM LBH589 by phase contrast microscopy, Alizarin red staining, and *β*-galactosidase staining, respectively. Scale bar = 500 *μ*m. (e) Soft agar clonogenicity assay of U2OS and SJSA human osteosarcoma cells pretreated with 15 nM LBH589 for 21 days. Mean ± SEM, *P < 0.05, and **P < 0.01.

To investigate the effect of LBH589 in osteosarcoma, we cultured a panel of 5 human osteosarcoma cell lines (U2OS, SJSA, Saso2, MG-63, and B143) in the presence of increasing concentrations of LBH589 (0.5–500 nM). Assessment of cell viability using alamarBlue demonstrated a significant reduction in cell growth of all cell lines following 96 hours of continuous exposure with an IC_50_ of approximately 6, 8, 3, 22, and 8 nM, respectively, (Figures 1(a) and 1(b), Supplementary Figure 1). Specifically, LBH589 concentrations <15 nM resulted in a marked reduction in cell growth, 15–30 nM caused a growth arrest, and >30 nM led to cell death as observed morphologically by cell rounding and detachment (Supplementary Figure  2A). Despite reduced cellular growth, no cell death was observed below 30 nM. Cell cycle analysis following 48 hours exposure to DMSO control, low-dose LBH589 (15 nM), and high-dose LBH589 (200 nM) demonstrated an accumulation of cells in G0/G1 (43% versus 48% and 70% resp.) and a reduced proportion of cells in S-phase (31% versus 24% and 12% resp.) consistent with reduced cellular growth and arrest (Supplementary Figure  2B). Analysis of apoptosis by Annexin V staining in these samples revealed a similar proportion of early apoptotic cells in DMSO (5%) and low-dose LBH589-treated (7%) cells but a marked increase in high-dose LBH589-treated (23%) cultures consistent with our morphological observations (Supplementary Figure  2C). To assess the effects of LBH589 on acetylation of histone proteins, the human osteosarcoma cell lines were cultured for 24 hours in the presence of increasing concentrations of LBH589 (5–200 nM). All cell lines demonstrated a progressive increase in histones H3 and histone H4 acetylation with increasing concentrations of LBH589 (Figure 1(c), Supplementary Figure  1). Similarly, acetylation of the nonhistone protein, *α*-Tubulin, also increased with increasing LBH589 concentrations (Figure 1(c), Supplementary Figure  1). Interestingly, acetylation of another nonhistone protein, P53, was only observed at high LBH589 concentrations associated with cell death (Figure 1(c)). Notably, the most dramatic increase in Histone protein acetylation occurred between 10 and 20 nM of LBH589, corresponding to the concentrations that elicit the most pronounced growth inhibition in the absence of cell death. Since there is also no detectable P53 acetylation at this range, we selected 15 nM to further investigate the mechanisms of action of a low-dose, sublethal concentration of LBH589 in osteosarcoma cells. 

### 3.2. Low-Dose LBH589 Induces Differentiation and Senescence of Human Osteosarcoma Cells

We investigated the consequence of sustained growth inhibition and arrest caused by continuous treatment of human osteosarcoma cell lines with 15 nM LBH589 over a 21-day culture period. Inhibition of osteosarcoma cell growth was preceded by an almost complete growth arrest in the U2OS, SJSA, Saos-2, and MG-63 cell lines after approximately 7 days of culture accompanied by a progressive change in cell morphology. In contrast to the small, spindle-shaped cells in DMSO control cultures, cells treated with 15 nM LBH589 were significantly larger with large extracellular projections (Figure 1(d), Supplementary Figure  1). These cells remained viable over the remaining duration of the culture period. To determine whether LBH589-mediated growth inhibition was irreversible, we washed out LBH589 and replaced with normal growth medium at daily intervals (Supplementary Figure  3). Whilst U2OS cells pretreated for 1–6 days with 15 nM LBH589 resumed a growth rate similar to DMSO controls, cells cultured for ≥7 days demonstrated a sustained growth inhibition following LBH589 withdrawal (Supplementary Figure  3). The dramatic growth arrest and distinct morphology of LBH589-treated cells suggested they had undergone terminal differentiation and/or cellular senescence. Since, osteosarcoma cells undergo osteoblast differentiation when cultured in osteogenic culture media [15], we investigated the possibility of low-dose LBH589 alone inducing osteoblast differentiation. In accord with this, cells treated with 15 nM LBH589 for 21 days stained positively with a marker of mineralized extracellular matrix, Alizarin Red (Figure 1(d)). Low-dose LBH589 also induced senescence of osteosarcoma cells as evidenced by **β*-galactosidase* staining following 21 days treatment (Figure 1(d)).

We reasoned that cell differentiation and senescence are at the expense of osteosarcoma cell self-renewal. Indeed, colony numbers of U2OS (101.3 ± 14.7 versus 32.0 ± 5.0, *n* = 3, *P* < 0.05) and SJSA (273.0 ± 11.0 versus 70.0 ± 7.0, *n* = 3, *P* < 0.01) cells were significantly reduced in soft agar following 15 nM LBH589 treatment for 21 days (Figure 1(e)). These results demonstrate that low-dose LBH589 reduces osteosarcoma cell clonogenicity by inducing senescence and differentiation of human osteosarcoma-initiating cells.

### 3.3. Low-Dose LBH589 Treatment of Osteosarcoma Cells Induces Changes in Relevant Gene Expression Profiles

**Figure 2:**
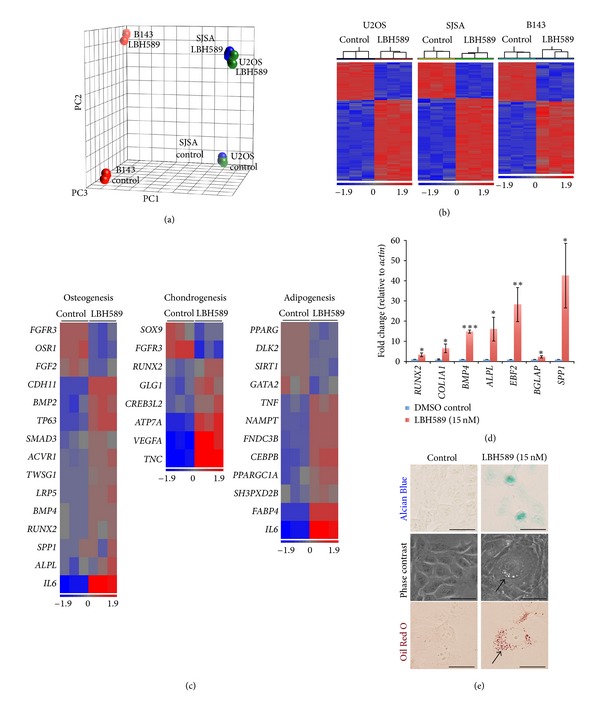
Microarray analysis of human osteosarcoma cell lines. (a) Principle component analysis (PCA). Three PCA coordinates describe 56% of the total data variation (PC1, 8.8%; PC2, 18.1%; PC3, 29.1%). Red, B143; blue, SJSA; green, U2OS. (b) Unsupervised hierarchical cluster analysis. Heatmap representation of differentially expressed genes in U2OS, SJSA, and B143 cells treated for 21 days with 15 nM LBH589 or DMSO. Each column represents a distinct sample, and each row represents an individual gene. Level of expression is denoted by colour (blue, low; red, high). (c) Heatmap representation of genes enriched in functional groups associated with osteogenesis, chondrogenesis, and adipogenesis. Each column represents a distinct sample. (d) Quantitative real-time PCR of U2OS cells cultured for 21 days in the presence of 15 nM LBH589 for expression of osteoblast differentiation markers. *P < 0.05, **P < 0.01, and ***P < 0.001. (e) Analysis of chondrocyte and adipocyte differentiations of human osteosarcoma cells treated for 21 days in 15 nM LBH589 by Alcian Blue staining, a marker of cartilage extracellular matrix, phase contrast microscopy, and the triglyceride marker, Oil Red O. Scale bar = 250 *μ*m.

To assess LBH589-induced changes in global mRNA expression changes, we performed genome-wide transcriptional profiling of U2OS, SJSA, and B143 cells following 21 days of continuous treatment with 15 nM LBH589. Principle component analysis of microarray data revealed a low level of variability among biological replicates and a marked separation of the control and LBH589 treatment groups for each cell line (Figure 2(a)). Further analysis of the U2OS, SJSA, and B143 microarray data by hierarchical cluster analysis also confirmed low variability (Figure 2(b)) and identified 1055 (337 downregulated and 718 upregulated), 1103 (336 downregulated and 767 upregulated) and 1711 (599 downregulated and 1112 upregulated) differentially expressed genes between DMSO control and 15 nM LBH589-treated cells, respectively (Supplementary Tables  2, 3, and 4). 

**Figure 3:**
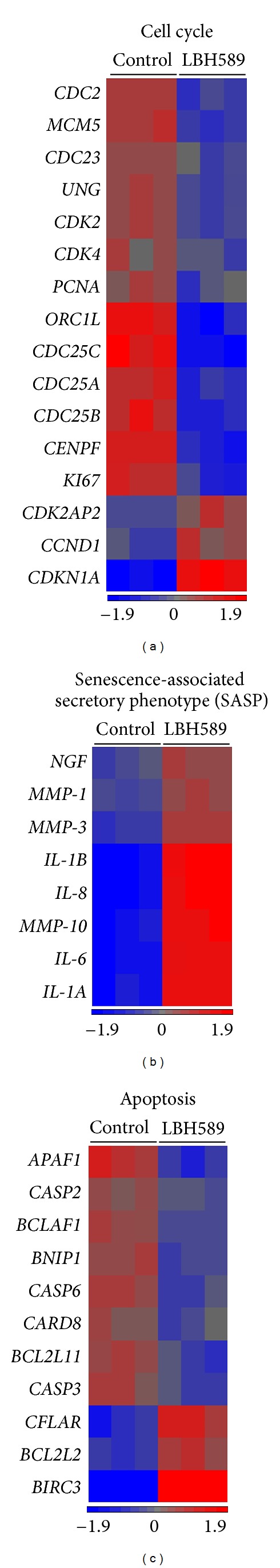
Low-dose LBH589 treatment induces cell cycle arrest and senescence. Heatmap representation of differentially expressed genes enriched in functional groups associated with (a) cell cycle regulation, (b) senescence-associated secretory phenotype, and (c) apoptosis. Each column represents a distinct sample.

A gene ontology analysis of the U2OS data performed to identify functional groups of differentially expressed genes (Supplementary Table  5) revealed genes involved in cell cycle regulation and differentiation, including osteogenesis (Figures 2(c) and 3). Inspection of osteogenesis-related functional groups for genes that have a functional requirement during osteoblast differentiation and are downregulated following LBH589 treatment identified genes associated with proliferation of osteoprogenitors (*FGF2*), suppression of osteoblast differentiation (*OSR1*), and negative regulation of bone development (*FGFR3*). In contrast, genes upregulated following LBH589 treatment included markers of osteoblast differentiation (*RUNX2, ALPL, BMP4,* and *SPP1*), positive regulators of skeletal and bone development or ossification (*TWSG1, SMAD3, TP63,* and *BMP2*), and genes expressed by mature osteoblasts (*IL6, LRP5,* and *CDH11*) (Figure 2(c)). To validate upregulation of osteogenesis-related genes, quantitative real-time PCR for a panel of known osteoblast differentiation makers revealed a significant increase in markers of the osteoblast precursor *RUNX2 *(3.2 fold ± 0.9, *P* < 0.05), preosteoblasts *COL1A1 *(6.5 fold ± 2.2, *P* < 0.05)*, BMP4 *(14.8 fold ± 0.8, *P* < 0.001)*, ALPL *(16.0 fold ± 5.9, *P* < 0.05)*, EBF2 *(28.2 fold ± 8.5, *P* < 0.01), and mature osteoblasts *BGLAP/*osteocalcin (2.3 fold ± 0.5, *P* < 0.05) and *SPP1*/osteopontin (42.5 fold ± 16.0, *P* < 0.05) (Figure 2(d)). Taken together these results suggest that low-dose LBH589 drives osteoblast differentiation of human osteosarcoma cells.

### 3.4. Differentiation of Alternative Mesenchymal Lineages Is Induced by LBH589

Other functional groups enriched by the gene ontology analysis included the alternative mesenchymal lineage pathways, chondrogenesis, and adipogenesis. Similar to the osteogenesis-related functional group, genes that were downregulated in the LBH589 group were associated with the negative regulation of chondrogenesis or adipogenesis, while genes that were upregulated strongly associated with positive regulation of these processes (Figure 2(c)). To further explore this, we stained 21-day cultured cells with Alcian Blue or Oil Red O, markers of cartilage extracellular matrix and lipid droplets, respectively (Figure 2(e)). Alcian Blue positive staining was observed in LBH589-treated cells but was completely absent in DMSO control-treated cultures, indicative of terminal chondrocyte differentiation (Figure 2(e)). Similarly, the presence of intracellular lipid droplets was confirmed in 15 nM LBH589 but not in DMSO control-treated cells by phase microscopy and positive Oil Red O staining, demonstrating terminal adipocyte differentiation (Figure 2(e)). These data are consistent with the possibility that the uncommitted mesenchymal progenitors are the cell of origin for osteosarcoma and further support the differentiation capabilities of low-dose LBH589. 

### 3.5. LBH589 Induces Cell Cycle Arrest and Senescence of Human Osteosarcoma Cells

The marked enrichment of functional groups associated with cell cycle regulation in our array data prompted us to explore this in more detail. Expression of proliferation markers *KI67* and *PCNA* were reduced following LBH589 treatment consistent with cell growth inhibition and arrest. Notably, genes required for G1/S transition were overrepresented in our array data, including *CDKN1A, CCND1, CDK2, CDK4, MCM5, CDC2, CDC25A,* and *CDC25C* (Figure 3) suggesting a cell cycle arrest in the G0/G1 phase. This gene expression profile is consistent with the cell cycle analysis following 48 hours of continuous LBH589 treatment demonstrating an increased proportion of cells in G0/G1 and reduced proportion of cells is S-phase (Supplementary Figure  2). G1 phase growth arrest and changes in cellular morphology are consistent with terminal cell differentiation [17] and/or cell senescence and also corroborate our data described above. Furthermore, our array results demonstrated an increase in genes associated with the secretory-associated senescence phenotype (SASP) (Figure 3). These results suggest that both cellular differentiation and senescence are features of low-dose LBH589-mediated growth arrest. Interestingly, whilst the P53 transcriptional target, *CDKN1A, *was upregulated >3-fold in the LBH589-treated cells, the absence of P53 acetylation (Figure 1(c)) is consistent with selective P53-independent induction *of CDKN1A* by HDACi [18]. Indeed, similar LBH589-responsiveness, growth arrest, and differentiation phenotypes in the P53-null Saos-2 and MG-63 human osteosarcoma cells (Supplementary Figure  1) strongly suggest that at least at low-dose LBH589 acts through P53-independent mechanisms.

Consistent with 15 nM LBH589 being a sublethal concentration based on the absence of morphological cell apoptotic features, no detectable loss of cell number, and similar proportion of early apoptotic cells to DMSO control following 48 hours culture, our gene expression data demonstrated increased expression of antiapoptotic genes (*BIRC3, BCL2L2,* and *CFLAR*) and decreased expression of proapoptotic genes (*CASP3, BCL2L11, CARD8, CASP6, BNIP1, BCLAF1, CASP2,* and *APAF1*) following LBH589 treatment (Figure 3).

### 3.6. LBH589 Reduces Osteosarcoma Tumour Growth **In Vivo**

**Figure 4:**
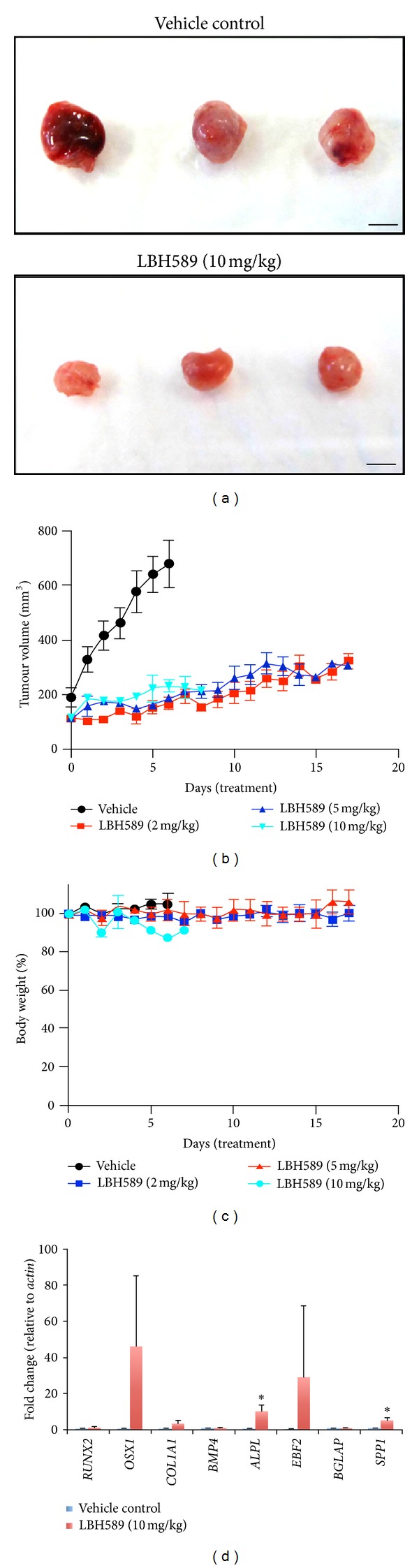
LBH589 reduces osteosarcoma tumour growth and drives osteoblast differentiation *in vivo*. (a) U2OS xenograft flank tumours treated with vehicle control or 10 mg/kg LBH589 i.p. daily for 6 days. Bar = 5 mm. (b) Tumour volume. Mean ± SEM. (c) Percentage body weight. Mean ± SEM. (d) Quantitative real-time PCR of U2OS xenograft tumours following 6 consecutive days of treatment with vehicle control for expression of osteoblast differentiation markers. *P < 0.05.

To investigate the therapeutic potential of LBH589 on osteosarcoma *in vivo*, 1 × 10^6^ U2OS cells were injected into the flanks of Nude mice. We observed 100% engraftment of cells with a tumour volume of ~150 mm^3^ reached 2-3 weeks after inoculation. Mice were initially randomized to receive vehicle control or 10 mg/kg LBH589 daily by intraperitoneal injection [19]. Maximum tolerated dose of LBH589 is 15 mg/kg qd based on a 15% of body weight loss at 10–14 days (Novartis, personal communication). Tumours in mice treated with vehicle control grew rapidly and reached endpoint size within 5-6 days following the commencement of treatment (Figures 4(a) and 4(b)). In contrast, tumours in mice treated with 10 mg/kg LBH589 were significantly smaller and demonstrated minimal growth over a 7-day treatment period (Figures 4(a) and 4(b)). After 5–7 days of treatment, the body weight of mice receiving 10 mg/kg LBH589 decreased by ≥10%, and the experiment was terminated due to LBH589 dose intolerance and ethical endpoints (Figure 4(c)). This is consistent with a previous study using human gastrointestinal stromal tumour xenografts that reported dose-limiting toxicities including 21% of body weight loss, skin dehydration, diarrhea, and histopathological changes in the spleen and small bowel following 12 days of continuous treatment with 10 mg/kg LBH589 [19]. To determine if LBH589 could also drive osteoblast differentiation of osteosarcoma cells *in vivo,* quantitative real-time PCR was performed on tumour tissue collected following 6 days of treatment with 10 mg/kg LBH589. Consistent with the *in vitro* cell culture findings, we observed increased expression of osteoblast-specific differentiation markers, including the preosteoblast marker *ALPL* (3.4 fold ± 1.9, *P* < 0.05) and mature osteoblast marker *SPP1* (4.9 fold ± 1.7, *P* < 0.05) indicating a differentiation phenotype (Figure 4(d)).

To improve LBH589 tolerance *in vivo*, we explored the outcome of lower LBH589 doses on tumour growth. Remarkably, 2 mg/kg and 5 mg/kg, representing a fifth and a half of the original LBH589 dose, respectively, did not result in any detectable adverse side effects despite a tumour response identical to the higher dose (Figures 4(b) and 4(c)).

## 4. Discussion

Despite the use of adjuvant chemotherapy, the prognosis for osteosarcoma patients with metastatic and/or recurrent disease remains poor, highlighting the urgent need for new and improved therapies. HDACis are an emerging class of anticancer agents with high activity in hematological malignancies. However, the underwhelming effect on solid malignancies and significant adverse side effects associated with doses required to elicit a desired cytotoxic effect have limited their applications. Here, we have identified that sub-lethal, low-dose LBH589 acts as a potent inducer of osteosarcoma cell differentiation. Culture of human osteosarcoma cell lines with low-dose LBH589 inhibits cell growth and clonogenicity, induces cell cycle arrest and senescence, and results in terminal differentiation into mature, bone forming, osteoblasts. LBH589 treatment of a mouse xenograft model of osteosarcoma resulted in a significant inhibition of tumour growth and increased the expression of osteoblast differentiation markers. Titration of the LBH589 dose demonstrated a similar tumour response in the absence of any detectable sign of toxicity.

### 4.1. Osteosarcoma Results from Abnormal Differentiation

Abnormal cellular differentiation is a characteristic of nearly all cancers, including osteosarcoma. It is proposed that two key transition points exist in normal osteoblast differentiation that are the focus of oncogenic events: (1) transition from the mesenchymal stem cell (MSC) to a osteoblast-restricted progenitor; (2) termination of osteoblast lineage expansion and progression of terminal differentiation [20]. Evidence exists to support both scenarios. First, consistent with our findings, osteosarcomas have the capacity for multilineage differentiation along mesenchymal lines. Second, osteosarcoma frequently exhibits expression of early osteogenic differentiation markers such as *RUNX2* and *OSX1* but not terminal differentiation markers, osteocalcin and osteopontin [1]. Third, genetic mouse models in which *Trp53* and *Rb* are conditionally mutated through targeted deletion under the control of the *Osx1* promoter develop osteosarcoma [21]. Since *Osx1* is a master regulator of preosteoblast lineage commitment, these experiments demonstrate that the osteoprogenitor lineage is competent to support tumor initiation. Regardless of the precise inflection point, greater than 80% of osteosarcomas are histopathologically graded as “poorly differentiated,” a feature associated with a 10–15% decrease in 5-year survival [20]. The ability of osteosarcoma cells to retain an undifferentiated phenotype permits uncontrolled proliferation and resistance of apoptosis. For these reasons, targeting differentiation defects in osteosarcoma present as an attractive therapeutic option. Indeed, a number of transcription factors, growth factors, and nuclear receptor agonists have been studied in the context of differentiating-promoting agents in osteosarcoma, but their nonspecificity and/or inability to pharmacologically target has failed to lead to translational outcomes (reviewed in [1]).

### 4.2. Epigenetic Regulation of Osteoblast Differentiation

Increasing evidence suggests the mechanisms underlying abnormal differentiation in osteosarcoma may be epigenetically regulated. During osteoblast development, acetylation of histones H3 and H4 increases accessibility of *Runx2* to osteogenic promoter regions, including osteocalcin [22]. Further, *Runx2* itself is directly suppressed by HDAC4, and absence of HDAC4 results in transcriptional activation and increased ossification [23]. Similarly, decreased methylation of the osteopontin promoter is associated with an increased expression and osteogenic differentiation [24]. More recently, in a landmark study, Kansara et al. identified several potential tumour suppressor genes to be epigenetically silenced in osteosarcoma [15]. Notably, *WIF1* is a known regulator of the WNT signaling pathway and critical coordinator of osteoblast proliferation and differentiation [15]. Together, these studies provide compelling data implicating epigenetic regulation in normal and abnormal osteoblast differentiation contributing to osteosarcoma.

### 4.3. HDACi in Osteosarcoma

HDACis have previously been proposed for osteosarcoma based on their ability to inhibit human and canine osteosarcoma cell growth by inducing apoptosis, primarily through Fas-mediated or caspase-dependent mechanisms [25–28]. Here, using sublethal concentrations of LBH589, we have identified what we believe to be a novel function of HDACi in inhibiting osteosarcoma growth via regulation of tumour cell differentiation. Interestingly, valproic acid and sodium butyrate promote preferential osteogenic differentiation of human mesenchymal stem cells [29]. Considering the pathogenic features of osteosarcoma discussed above, it is tempting to speculate that this disease is particularly sensitive to epigenetic-driven differentiation. 

## 5. Conclusion

The differentiating potential of HDACi was initially described in murine erthyroluekemia cells in 1975 [11, 12]. More recently, the ability of suberoylanilide hydroxamic acid (SAHA/vorinostat) and trichostatin A to induce mammary gland differentiation of human breast cancer cells suggests that solid tumours are also susceptible to HDACi-dependent differentiation [17, 30]. Despite these findings, this potential mechanism of function and therapeutic strategy for HDACi has been largely ignored in the cancer setting in favor of cytotoxic outcomes. However, the clinical advancement of HDACi in solid tumours has been impeded by poor tumour response, in terms of regression and associated adverse side-effects [13]. Here, we have reported that low-dose LBH589 inhibits osteosarcoma cell growth by driving tumor cell differentiation, rather than death. While the precise mechanism by which HDACi is able to promote differentiation at low-dose and apoptosis and high-dose remains to be determined, we propose that low-dose LBH589 acts predominantly as a potent “differentiating” agent. Consequently, LBH589 may provide an effective and well-tolerated therapeutic option for the treatment of osteosarcoma and likely other solid tumours, particularly with an undifferentiated phenotype.

## Supplementary Material

Supplementary Figure 1: Effect of LBH589 in human osteosarcoma cells. Cell viability, western blot analysis and cell morphology in human osteosarcoma cells following 21 days culture in 15nM LBH589. A, B143 cell line. B, MG-63 cell line. C, Saos-2 cell line. D, SJSA cell line. Bar = 500 *μ*m.
Supplementary Figure 2: Effect of LBH589 in human osteosarcoma cells on cell cycle and apoptosis. A, Phase contrast microscopy. B, Analysis of cell cycle by flow cytometry. C, Analysis of apoptosis by flow cytometry. Early apoptotic cells are represented by PI negative, Annexin V positive expression (bottom right quadrant). Bar = 500 *μ*m.
Supplementary Figure 3: LBH589-mediated growth inhibition is irreversible. Cell viability following withdrawal of 15nM LBH589.
Supplementary Table 1: Primers for quantitative real-time PCR.
Supplementary Table 2: U2OS differentially expressed genes following 21-days culture (DMSO vehicle vs 15nM LBH589).
Supplementary Table 3: SJSA differentially expressed genes following 21-days culture (DMSO vehicle vs 15nM LBH589).
Supplementary Table 4: B143 differentially expressed genes following 21-days culture (DMSO vehicle vs 15nM LBH589).
Supplementary Table 5: U20S gene ontology analysis.
