# Supplementary material for: Sustained Low-Dose Treatment with the Histone Deacetylase Inhibitor LBH589 Induces Terminal Differentiation of Osteosarcoma Cells
Source: Sarcoma. 2013 Feb 28;2013:608964. doi: 10.1155/2013/608964 (PMC3603321; doi:10.1155/2013/608964)
Supplement: Supplementary file 1 — Supplementary Figure 1: Effect of LBH589 in human osteosarcoma cells. Cell viability, western blot analysis and cell morphology in human osteosarcoma cells following 21 days culture in 15nM LBH589. A, B143 cell line. B, MG-63 cell line. C, Saos-2 cell line. D, SJSA cell line. Bar = 500 μm. Supplementary Figure 2: Effect of LBH589 in human osteosarcoma cells on cell cycle and apoptosis. A, Phase contrast microscopy. B, Analysis of cell cycle by flow cytometry. C, Analysis of apoptosis by flow cytometry. Early apoptotic cells are represented by PI negative, Annexin V positive expression (bottom right quadrant). Bar = 500 μm. Supplementary Figure 3: LBH589-mediated growth inhibition is irreversible. Cell viability following withdrawal of 15nM LBH589. Supplementary Table 1: Primers for quantitative real-time PCR. Supplementary Table 2: U2OS differentially expressed genes following 21-days culture (DMSO vehicle vs 15nM LBH589). Supplementary Table 3: SJSA differentially expressed genes following 21-days culture (DMSO vehicle vs 15nM LBH589). Supplementary Table 4: B143 differentially expressed genes following 21-days culture (DMSO vehicle vs 15nM LBH589). Supplementary Table 5: U20S gene ontology analysis. [file 608964.f1.zip › Table S1.docx]

**Supp Table 1.** Primers for quantitative real-time PCR.

**mRNA Forward Primer Reverse Primer**

*RUNX2* ggtcccttcattggaatcct gcctacaaaggtgggtttga

*OSX1* ggcacaaagaagccgtactc caggtgaaaggagcccatta

*COL1A1*  ggcccagaagaactggtaca aatccatcggtcatgctctc

*BMP4*  tccacagcactggtcttgag atgttcttcgtggtggaagc

*ALPL* ggacatgcagtacgagctga cagcaagaagaagcctttgg

*EBF2*  ggagtggtggacgctaatgt tcaagttggaaggaggctgt

*BGLAP* (Osteocalcin) gactgtgacgagttggctga ctggagaggagcagaactgg

*SPP1* (Osteopontin) ggacaaggctacgatggcta gatgcaaagccagaatggat
